# How Stable are Moral Judgments?

**DOI:** 10.1007/s13164-022-00649-7

**Published:** 2022-07-29

**Authors:** Paul Rehren, Walter Sinnott-Armstrong

**Affiliations:** 1 Ethics Institute, Department of Philosophy and Religious Studies, Utrecht University, Utrecht, The Netherlands; 2 Department of Philosophy, Duke University, Durham, NC United States of America

**Keywords:** Moral judgment, Stability, Sacrificial dilemmas, Longitudinal

## Abstract

Psychologists and philosophers often work hand in hand to investigate many aspects of moral cognition. In this paper, we want to highlight one aspect that to date has been relatively neglected: the stability of moral judgment over time. After explaining why philosophers and psychologists should consider stability and then surveying previous research, we will present the results of an original three-wave longitudinal study. We asked participants to make judgments about the same acts in a series of sacrificial dilemmas three times, 6–8 days apart. In addition to investigating the stability of our participants’ ratings over time, we also explored some potential explanations for instability. To end, we will discuss these and other potential psychological sources of moral stability (or instability) and highlight possible philosophical implications of our findings.

Psychologists and philosophers often work hand in hand to investigate many aspects of moral cognition (see, Sinnott-Armstrong 2008c, 2008b, 2008d, 2014; Sinnott-Armstrong and Miller 2017). One issue has, however, been relatively neglected: the stability of moral judgments over time.

This paper will begin to address that lacuna. We will first explain why stability is important to philosophers and psychologists. Then we will survey previous research on the stability of moral judgments. Next, we will present the results of an original empirical study. We will end by discussing potential psychological sources of moral stability (or instability) and philosophical implications of our findings.

## Why does Stability Matter?

There are four main reasons for philosophers and psychologists to consider the stability of moral judgments over time.

First, the stability of moral judgments over time can shed light on the role of moral values in moral judgments. Many psychologists agree that moral values1 structure moral judgment and decision-making in a stable way, in the sense that they anchor moral judgments and decisions across a variety of different situations (e.g., Feather 1995; Hitlin and Piliavin 2004; Rohan 2000; Schwartz 2012; Tetlock 1986). For example, if someone judges that it is morally wrong to lie because honesty is a moral value, then they should continue to value honesty and think that it is wrong to lie in different contexts where the lie is about a variety of topics and the lie is told to strangers, friends or colleagues. Similarly, moral philosophers often stress that moral judgments should be based on more fundamental moral values in a stable way (see, Audi 2007, Chap. 1).

The stability of moral judgments matters here, because if moral judgments are often not stable over time, then this speaks against the idea that moral values generally structure moral judgments in a stable way. If moral values do structure people’s judgments in a stable way, then people should at least make the same moral judgments when they judge *exactly the same* scenario or statement on multiple occasions. Conversely, if moral judgments about the *same* scenarios or statements do not remain stable, even over a short period of time (days or weeks), then this would provide evidence against the claim that people’s moral judgments are generally structured in a stable way by moral values (see, Helzer et al. 2017, pp. 2–3).

Why should moral philosophers care? Moral theory is not about how things are, but about how things should be, and so even if in the real world, moral judgments are not generally based in a stable way on moral values, what does it matter? It matters because most moral theorist do not see their work as a purely speculative exercise, completely divorced from the real world. Instead, moral theory is supposed to help real people lead better, more moral lives. If so, then it is important to make sure that moral theory does not assume a moral psychology that is too distant from the moral psychology of real human beings (cf., Anscombe 1958). Otherwise, it may be doubtful whether moral theory will be successful in realizing its practical ambitions (see, Flanagan 1991, Chap. 1).

Second, lay people seem to expect moral judgments to be stable in a way that contrasts with tastes. Many diners order pasta one day and pizza the next. Nobody criticizes them for changing what they prefer to eat, even if they have no reason to change. In contrast, if someone says on one day that capital punishment is immoral and then says on the next day that it is not immoral, then we suspect that this person would be subject to criticism as inconsistent, at least if they had no good reason to change their moral judgment. This suspicion is borne out by empirical studies. For example, Kreps and Monin (2014; also, see Skitka et al. 2015) find that decisions which are supposed to be based on moral as opposed to non-moral concerns are seen as more committal and less flexible. In a similar vein, leaders who change their opinion after having taken a moral stance are perceived as hypocritical, ineffective, and unworthy of support (Kreps et al. 2017). Thus, people seem to expect people’s moral decisions and judgments to be based on stable moral values that do not change arbitrarily across time.

Admittedly, an important enough difference between the acts that are judged or the circumstances of those acts can justify making different moral judgments. There is nothing objectionable with judging that one promise ought to be broken, but another promise ought to be kept, if keeping the former will cause great harm, whereas keeping the latter will not hurt anyone. Nonetheless, it is typically seen as objectionable to say that a promise ought to be kept in certain circumstances and then later say that the very same promise ought to be broken in the very same circumstances, at least when one did not learn anything new about the promise or the situation. Incompatible moral judgments about the same action in the same circumstances cannot be justified by any difference between what is judged on the two occasions.

Third, philosophers also assume that *their* moral judgments do and should not change without reason. They often test general moral theories by appealing to moral judgments about particular actions in scenarios (though see, Deutsch 2010; Williamson 2007). Such responses have been called the “data of ethics” (Ross 1930/2002, p. 41). The most widely used method of this sort is reflective equilibrium (Kamm 1993; Rawls 1971). It consists in “working back and forth among our considered judgments […] about particular instances or cases, the principles or rules that we believe govern them, and the theoretical considerations that we believe bear on accepting these considered judgments, principles, or rules, revising any of these elements wherever necessary in order to achieve an acceptable coherence among them” (Daniels 2020). This “acceptable coherence” cannot be achieved when we make incompatible moral judgments about the very same action in the very same circumstances. Moral theories, therefore, cannot be justified by these methods unless they appeal to moral judgments that are stable in this way.

One problem with unstable moral judgments is that they are not reliable in the sense of likely to be correct (in a sense of “correct” that is consistent with moral realism or quasi-realism). Incompatible moral judgments about the same act in the same circumstances cannot both be correct. That is what it means to call them “incompatible.” Thus, when an individual makes a moral judgment at one time but then makes (or would make) an incompatible judgment at a different time about the same act in the same scenario, then these two moral judgments cannot both be correct. If we have no independent way to tell which of the two moral judgments is correct, then we cannot determine which one—if either—is trustworthy. If these are the “data of ethics” (Ross, 1930/2002, p. 41), then instability would raise serious doubts about the data.

This problem resembles interpersonal disagreements when neither person can be dismissed as less competent or reliable. Many philosophers have argued that we should not trust judgments about which epistemic peers disagree (see, Frances and Matheson 2019). An individual who endorses incompatible moral judgments on different occasions effectively disagrees with themselves over time. And if the same person makes both judgments, then there is no reason to dismiss either person or either judgment as incompetent or unreliable, unless some special feature of the circumstances in which one of the judgments is made is known to distort moral judgments. Instability then challenges the trustworthiness of moral judgments at least as much as interpersonal disagreement does.

Finally, stability also may have methodological implications for moral psychology. If someone were to tell a woman that she ought to stay at home with her kids and not pursue a career as a scientist, then many lay people would take this moral judgment to show that the person who made it has a character trait of sexism. Similarly, psychologists sometimes ask study participants to make moral judgments, and then the psychologists draw conclusions about their participants’ individual differences or traits. For example, some researchers who investigate whether people are utilitarians measure this using moral judgments about sacrificial dilemmas (for a review, see, Kahane et al. 2018, pp. 136–137). However, if people make incompatible moral judgments on different occasions without any reason for the change, then their moral judgments on only one occasion (or even a few occasions) cannot be strong evidence for any lasting individual difference or personality trait. Instead, people whose judgments change arbitrarily seem to lack any underlying moral values that structure, guide, or cause their judgments. If too many people display too much of this kind of instability in their moral judgments, then psychologists who try to discover underlying moral traits and values are bound to fail.

In these ways, the stability of moral judgments over time is essential to popular views and assumptions in both moral philosophy and moral psychology. However, these problems arise only if many people’s moral judgments actually do change from occasion to occasion without reason. So, the next question is: To what extent do human moral judgments display such arbitrary instability?

## Previous Studies of Stability in Moral Judgments

To our knowledge, only one study so far has directly addressed this question. Helzer et al. (2017, Study 1) asked participants to read and rate (‘Yes’/‘No’) the moral appropriateness of five actions in sacrificial dilemmas, which are scenarios in which an agent must sacrifice something valuable to prevent the loss of something else of value. In a classic example, an out-of-control trolley is speeding towards five people. The only way to save them is to divert the trolley onto a separate track by hitting a switch. However, there is a sixth person on this other track who will then be run over and killed by the trolley if it is diverted (Foot 1967). Moral psychologists have used sacrificial dilemmas extensively in many surveys and experiments (Christensen and Gomila 2012; Mudrack and Mason 2013).

Helzer et al. then asked some of the same participants to rate the same series of dilemmas again after a short time (at least 8 days). To assess stability, Helzer et al. calculated the correlation between participants’ average responses in the first and second session. They report *r* = .66, and interpret this as evidence that “participants’ aggregated judgments were highly consistent” (p. 4).

Other sources of evidence about stability are studies of reliability of instruments that measure moral judgments. When psychologists measure a construct that is assumed to be consistent across time, they need to make sure that their instrument reflects this stability. To do so, psychologists often assess the instrument’s *test-retest reliability* by calculating the correlation between scores on the instrument at two different points in time (see, Domino and Domino 2006, pp. 43–44).

One well-known family of instruments involving moral judgments comes from the research tradition of Kohlberg and colleagues (e.g., Kohlberg 1971; Levine et al. 1985), who were interested in measuring people’s level of moral reasoning. One way they did this was to ask people to make moral judgments about a variety of different scenarios and moral dilemmas. The reported test-retest reliabilities of such instruments range from low, for example, *r* = .44 for the *Moral Judgment Interview* (Rubin and Trotter 1977), to high, for example, *r* = .72 for the *Sociomoral Reflection Measure—Short Form* (Basinger et al. 1995).

Another instrument, which has been influential recently, is the *Moral Foundations Questionnaire* (MFQ; Graham et al. 2011). The MFQ asks respondents to indicate their agreement with a series of short general moral statements in order to assess how much they endorse five psychological foundations of morality posited by Moral Foundations Theory (Harm, Fairness, Loyalty, Authority and Purity; Haidt and Graham 2007). These general moral statements are distinct from moral judgments about particular scenarios, but it still might be relevant to note how much stability was found in participants’ agreement with general statements. Graham et al. (2011, p. 371) found high test-retest correlations for all five moral foundations (Harm: *r =* .71, Fairness: *r =* .68, Loyalty: *r =* .69, Authority: *r =* .71, Purity: *r =* .82), while the correlations reported by Curry et al. (2019, p. 116) were lower (Harm: *r =* .51, Fairness: *r =* .46, Loyalty: *r =* .62, Authority: *r =* .59, Purity: *r =* .75).

While interesting, it is hard to draw conclusions from these results about many of the issues and questions we highlight above. In particular, this is true for those issues that will likely most interest moral philosophers. Psychometricians often consider *r*s *>* 0.70 adequate test-retest correlations (e.g., Domino and Domino 2006, p. 43). However, as psychometricians are also quick to point out, this is only a convention, nothing more. Does a test-retest correlation below 0.70 mean that the moral judgments in question are not based on stable moral values or that philosophers should not trust them or that psychologists should not draw conclusions about individual differences or personality traits from them? We find this difficult to say. Hence, another way of quantifying the stability of moral judgments over time may be useful in order for philosophers to get the most out of empirical research into this phenomenon.

A second limitation of these studies is that they do not tell us anything about why moral instability occurs, when it occurs. However, as we said, philosophers need not be worried by moral instability if it is due to people changing their mind in light of reasons, perhaps found by intervening reflection or discussion with other people. Philosophers often stress the importance of revising one’s moral judgments in light of reasons, so philosophers would likely welcome evidence of instability due to reasons. Moreover, research suggests that such revisions in light of reasons do occur. For example, when people are presented with a compelling counter-argument against one of their moral beliefs, they will sometimes change their mind (Paxton et al. 2012; Paxton and Greene 2010; Schwitzgebel et al. 2020). Likewise, when people realize that a moral judgment is incompatible with a general moral principle that they endorse, they will sometimes change their mind about the moral judgment (Horne et al. 2015). So, empirical research about the stability of moral judgments likely has different philosophical implications depending on whether instability is due to reasoned changes of mind or to something else.

## Our Study

In this article, we report the results of a three-wave longitudinal study that improves on Helzer et al. (2017, Study 1) in two ways. Study 1a reports the results of the first two waves. Participants read and made judgments about the same acts in a series of sacrificial dilemmas (6–8 days apart). Study 1b asked the same participants back for a third time in order to investigate whether participants changed their moral judgments between waves in Study 1a because they changed their minds in light of reasons (perhaps after they thought more about the scenarios or discussed them with others).

## Study 1a

### Method

#### Design

The study consisted of two waves, W1 and W2, separated by 6–8 days. In each wave, we asked participants to read and make moral judgments about the same acts in the same series of scenarios.

#### Materials

*Scenarios.* We chose six sacrificial dilemmas that are commonly used in research on moral judgment and decision making (for a review, see, Christensen and Gomila 2012). Scenarios varied in content but were matched closely in length and structure. For each scenario, we asked participants whether the agent should carry out an action that would sacrifice (cause the death) of one person so that five others can live. Participants indicated their answer on a seven-point scale labeled at both endpoints, from “Definitely should do it” (= 1) to “Definitely should not do it” (= 7). The midpoint of the scale (4) was labeled “Neither”.

#### Participants

Participants were recruited through the online subject pool Prolific (https://www.prolific.co). We restricted participation to current residents of the US and the UK whose first language was English and who had at least a 95% acceptance rating on Prolific.

Sample size was determined before any data analysis. For each scenario, our main quantity of interest was the proportion of participants who gave ratings on opposite sides of our scale midpoint in W1 and W2. In pilot research, the largest proportion we found for any scenario was 17.0%. We therefore aimed for a sample size large enough for us to detect proportions of 20.0% within a 95% Wilson score confidence interval (Agresti and Coull 1998) of size ≤ 10.0%. Power analysis (Vallejo et al. 2013) indicated that we needed a minimum sample size of 282. To allow for attrition and exclusions, we recruited an initial sample of *n* = 461 (307 female, 1 not specified; *M*(*SD*) = 32.8(12.4) y; 24.7% students; 93.3% UK nationals).

We excluded 67 participants who failed an instructional attention check (Oppenheimer et al. 2009) in W1. 356 participants returned for W2, of whom 68 failed the attention check. The final sample thus included *n* = 288 participants (201 female; *M*(*SD*) = 33.1(12.2) y; 25.0% students; 95.1% UK nationals).

#### Procedure

Participants were contacted by Prolific with an invitation to participate in the study, which was hosted on LimeSurvey (https://www.limesurvey.org). Participants gave informed consent, received instructions, and then completed the scenarios. In W1, we randomized the presentation order of scenarios for each participant. This order was then repeated in W2. To make it less likely in W2 that participants would remember already having read the scenarios a week earlier, thereby potentially introducing experimenter demand (cf., Aczel et al. 2018), we changed the names of the agents between waves, matching them in gender, ethnicity and length.

Participants were compensated $1.00 per wave.

#### Analysis

All analyses reported in this paper were carried out in R (R Core Team 2020). We begin by calculating test-retest reliability coefficients for ratings given in W1 and W2. To get the overall test-retest reliability, these coefficients were then transformed to Fisher *z* coefficients, averaged, and back-transformed (Silver and Dunlap 1987). Table 1 shows the results.

We use four additional measures of the stability of moral judgments over time. We define a *rating shift* as any change in rating between W1 and W2. We define a *rating reversal* as a rating shift that crosses the scale-midpoint (= 4). We define a *rating rejection* as a rating shift onto the scale-midpoint. Finally, we define a *rating adoption* as a rating shift away from the scale-midpoint. Table 1 shows the proportions of rating shifts, reversals, adoptions and rejections for each scenario. It also shows the mean rating change magnitudes (that is, the absolute value of the mean difference between W1 and W2 for each type of rating change, when it occurred) corresponding to each of these proportions for each scenario.

**Table 1:** Pearson’s product-moment correlation coefficients, proportions and mean magnitudes of rating shifts, reversals, adoptions and rejections for our sacrificial dilemmas between W1 and W2; square brackets show 95% confidence intervals, round brackets standard deviations

	r		Shifts	Reversals	Adoptions	Rejections
**Shark**	0.62 [0.54, 0.69]	Prop.	0.55 [0.49, 0.61]	0.16 [0.12, 0.21]	0.05 [0.03, 0.08]	0.06 [0.03, 0.09]
		Mean magnitude	1.89 (1.2)	3.43 (0.89)	2.14 (0.86)	1.31 (0.6)
**Gas**	0.55 [0.47, 0.63]	Prop.	0.56 [0.5, 0.62]	0.2 [0.16, 0.26]	0.07 [0.05, 0.1]	0.08 [0.05, 0.11]
		Mean magnitude	2.15 (1.29)	3.39 (1.11)	1.8 (0.89)	1.73 (0.88)
**Footbridge**	0.72 [0.66, 0.77]	Prop.	0.48 [0.42, 0.53]	0.1 [0.07, 0.14]	0.06 [0.04, 0.1]	0.06 [0.03, 0.09]
		Mean magnitude	1.8 (1.17)	3.4 (1.3)	1.72 (0.83)	1.81 (0.83)
**Transplant**	0.61 [0.54, 0.68]	Prop.	0.3 [0.25, 0.35]	0.08 [0.05, 0.11]	0.02 [0.01, 0.05]	0.02 [0.01, 0.04]
		Mean magnitude	2.12 (1.63)	4.55 (1.3)	1.43 (0.53)	2.33 (0.52)
**Expedition**	0.66 [0.59, 0.72]	Prop.	0.55 [0.49, 0.61]	0.16 [0.12, 0.21]	0.07 [0.05, 0.11]	0.06 [0.04, 0.09]
		Mean magnitude	1.85 (1.23)	3.22 (1.36)	1.76 (0.83)	1.53 (0.51)
**Baby**	0.74 [0.68, 0.79]	Prop.	0.51 [0.45, 0.57]	0.12 [0.09, 0.16]	0.09 [0.06, 0.13]	0.05 [0.03, 0.08]
		Mean magnitude	1.79 (1.17)	3.35 (1.23)	1.6 (0.76)	1.8 (0.94)
**Overall**	0.66 [0.58, 0.72]	Prop.	0.49	0.14	0.06	0.05
		Mean magnitude	1.93 (1.28)	3.56 (1.2)	1.74 (0.79)	1.75 (0.72)

**Fig. 1:**
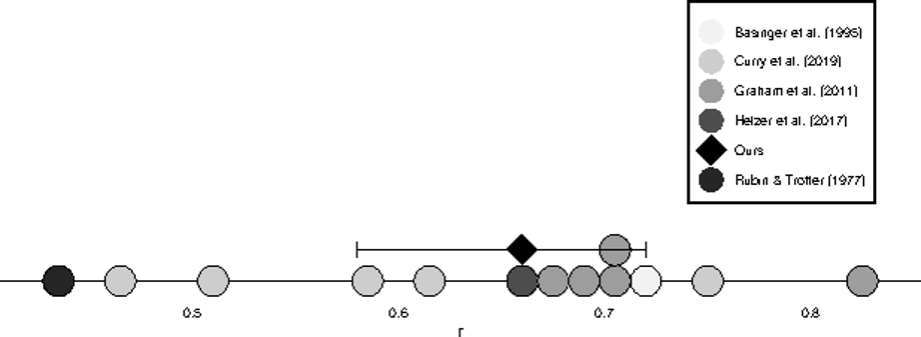
Comparison of our results with a sampling of test-retest studies of other instruments involving moral judgments (see, Previous Studies of Stability in Moral Judgments). Error-bars show 95% confidence intervals

Sacrificial dilemmas may lead to instability because these scenarios deliberately pit different moral concerns against one another; moral judgments about such conflicts might be less stable than many other moral judgments. To probe this possibility, we compared our results with the test-retest correlations found in previous work that we sampled in the introduction (Fig. 1). Figure 1 reveals that our results replicate the findings by Helzer et al. (2017, Study 1): both they and we found an overall correlation coefficient of *r* = .66. Moreover, this correlation is comparable to and does not seem to differ systematically from a sampling of test-retest correlations previously reported for other instruments involving moral judgments and general moral statements. This provides some evidence against the suggestion that moral judgments about acts in sacrificial dilemmas are atypical in terms of their instability over short periods of time.

Another possible explanation for instability is that participants were more confident in their moral judgments the second time around, and so in the second wave, many of them gave more extreme and therefore different ratings. While we did not measure confidence directly, we can still at least probe this explanation. First, higher confidence in the second wave likely cannot explain rating reversals, because in the case of rating reversals, ratings change polarity, not just degree. Likewise, higher confidence in the second wave cannot explain rating rejections, because here, participants go from judging that the agent should or should not do the act described in the scenario to sitting on the fence (responding “Neither”). The explanation may work for rating adoptions, because here, participants go from sitting on the fence to judging that judging that the agent should or should not do the act described in the scenario. Note, however, that rating adoptions only make up about 5% of all rating shifts.

What about the remaining rating changes? We would expect to see that ratings tend to get more extreme in W2 compared to W1. That is, participants who start out with a rating below (above) the scale midpoint in W1 will tend to give a lower (higher) rating in W2 than in W2; in other words, a rating closer to the extreme end of the rating scale. To test investigate this, we can calculate the proportion of rating changes that are not reversals, adoptions or rejections where the rating given in W2 was more extreme in than the rating given in W1. This proportion is 0.45 [0.40, 0.50]. Because this value is close to 0.50 (the 95% CI includes it), our data does not suggest that rating changes other than perhaps rating adoptions can be explained because participants are more confident in their moral judgments the second time around.

Another interesting question is whether rating changes when they occur are due mostly to a subset of participants changing their rating on many scenarios, or instead are due to a larger subset of participants changing their rating on a small number of scenarios. To investigate this, we calculated the proportion of participants who exhibited ratings shifts, reversals, adoptions and rejections on a given number of scenarios (zero to six). Table 2 shows the results.

**Table 2:** Proportions of people who exhibited ratings shifts, reversals, adoptions and rejections on a given number of scenarios (zero to six); square brackets show 95% confidence intervals

	Shifts	Reversals	Adoptions	Rejections
**0**	0.05 [0.03, 0.08]	0.46 [0.4, 0.52]	0.74 [0.68, 0.78]	0.74 [0.69, 0.79]
**1**	0.14 [0.1, 0.18]	0.33 [0.28, 0.39]	0.19 [0.15, 0.24]	0.21 [0.17, 0.26]
**2**	0.2 [0.16, 0.26]	0.16 [0.12, 0.2]	0.06 [0.03, 0.09]	0.05 [0.03, 0.08]
**3**	0.21 [0.17, 0.26]	0.04 [0.02, 0.07]	0.01 [0, 0.03]	0.01 [0, 0.02]
**4**	0.25 [0.2, 0.3]	0.01 [0.01, 0.04]	---	---
**5**	0.14 [0.1, 0.18]	---	---	---
**6**	0.01 [0.01, 0.04]	---	---	---

If rating changes when they occur are due to a smaller subset of participants who changed their rating on many scenarios, we would expect Table 2 to show that most of the participants with at least one rating change changed their rating on many scenarios (three or more). Conversely, if rating changes when they occur are due to a larger subset of participants changing their rating on a smaller number of scenarios, then we would expect to see that most of the participants with at least one rating change changed their rating on only one or two scenarios. For rating reversals, adoptions and rejections, the results in Table 2 better fit the first pattern. For rating shifts, the results in Table 2 are ambiguous between the two patterns. Overall, our results suggest that the rating changes we observed were not generally due to a small subset of participants changing their rating on many scenarios, but instead were due to a larger subset of participants changing their rating on a small number of scenarios.

## Discussion

In our study, we asked participants to respond to the same series of six sacrificial moral dilemmas twice, 6–8 days apart. Correlations between ratings in the first and second wave ranged from 0.55 to 0.74 (*M* = 0.66), the same value reported by Helzer et al. (2017, Study 1). We find that depending on the scenario, between 30% and 56% of participants exhibited a rating shift (*M* = 49%), with the mean magnitude of these shifts being close two full points (1.93). Between 8% and 20% of participants shifted such that their rating crossed the scale midpoint (*M* = 14%). The mean magnitude of these rating reversals was 3.56; that is, slightly more than half of the entire seven-point scale. In addition, between 2% and 9% of participants shifted onto the midpoint (*M* = 6%); between 2% and 8% shifted away from the midpoint (*M* = 5%). The mean magnitudes of these shifts were 1.74 and 1.75, respectively.

To probe the possibility that moral judgments about sacrificial dilemmas are more unstable than most other moral judgments, we compared our results with a sampling of test-retest studies of other instruments involving moral judgments and general moral statements (see, Previous Studies of Stability in Moral Judgments). In this comparison, our overall test-retest correlation did not stand out, which provides some evidence against the suggestion that moral judgments about acts in sacrificial dilemmas are atypical in terms of their instability over short periods of time.

Next, we investigated whether participants changed their rating because they were more confident in their moral judgments the second time around. While we did not measure confidence directly, we reasoned that in this case, participants should tend to give more extreme ratings in the second wave than in the first wave. However, our results did not support the idea that many rating changes (other than perhaps rating adoptions) can be explained because participants are more confident in their moral judgments the second time around.

Finally, we investigated whether the rating changes we observed were due to only a few participants changing their rating on many scenarios, or instead to most participants changing their rating on a small number of scenarios. To do this, we calculated the proportions of people who exhibited ratings shifts, reversals, adoptions and rejections on a given number of scenarios, and compared these proportions to the distributions we might expect for the two different options. Our results suggest that the rating changes we observed are better explained in terms of many participants changing their rating on a few scenarios, instead of few participants changing their rating on many scenarios.

## Study 1b

Another possible explanation of the results in Study 1a is that many of the observed changes between waves were due to our participants changing their mind in light of reasons, perhaps found by intervening reflection or discussion with other people. Study 1b asks how much of the observed changes of moral judgment between waves in Study 1a can be explained by participants changing their mind in light of reasons. To this end, we use three approaches. First, we look at the persistence of changes. If someone changes their moral judgment in light of reasons, then we would generally expect this change to last (Horne et al. 2015, p. 1953). Therefore, if changes between the first two waves do not tend to persist when a dilemma is judged for a third time, then this would provide some evidence that these changes did not occur in light of reasons.

A second method is to look at individual differences in whose moral judgments change between waves. If most changes of judgment occur in light of reasons, then participants who are generally more open to reconsidering and revising their judgments should be more likely to exhibit these changes. To measure this tendency, we here use the Actively Open-minded Thinking Scale (AOT; Baron 1993), which was designed to measure “the cultivation of reflectiveness rather than impulsivity, the seeking and processing of information that disconfirms one’s belief […], and the willingness to change one’s beliefs in the face of contradictory evidence” (Stanovich and West 1997, p. 346). Similarly, participants who tend to approach judgment and decision-making situations more rationally and reflectively should be more likely to exhibit these changes. To measure this second tendency, we use the Need for Cognition Scale (NFC; Cacioppo and Petty 1982), which was designed to measure “the tendency to engage in and enjoy cognitively effortful activities across a wide range of domains” (Petty et al. 2009, p. 319); and the Cognitive Reflection Test (CRT; Frederick 2005), which was designed to measure “the tendency to override a prepotent response alternative that is incorrect and to engage in further reflection that leads to the correct response” (Toplak et al. 2014, p. 1).

A third way to test the reasons hypothesis is through self-report. To the extent that changes in moral judgment between waves can be explained by reasons, this should be reflected in the number of participants who report having changed their minds about our sacrificial dilemmas either because they thought more about them or because they discussed them with others.

### Method

#### Design

For Study 1b, we invited all participants who previously completed W1 and W2 back for a third wave (W3), again after 6–8 days. We asked participants to again read and make moral judgments about the same series of scenarios from Study 1a. Moreover, participants completed several individual difference measures, answered self-report questions, and provided (additional) demographic information.

#### Materials

*Scenarios.* Same as in Study 1a.

*Actively Open-minded Thinking*. The original scale consists of 41 items. We here use a 10-item short form (AOT-10; Baron 2019). Items include “People should take into consideration evidence that goes against conclusions they favor” and “When we are faced with a new question, the first answer that occurs to us is usually best” (reverse coded). For each item, participants indicate their response on a five-point scale from “Strongly agree” (= 1) to “Strongly disagree” (= 5), with the midpoint labeled “Neither agree nor disagree” (= 3). Cronbach’s alpha was 0.68, comparable to α = 0.75 reported by Baron (2019).

*Need for Cognition.* The original scale consists of 34 items. Here, we are using a five-item short form (NFC-5; Epstein et al. 1996). Items include “I prefer complex to simple problems” (reverse coded) and “I don’t like to have to do a lot of thinking.” For each item, participants indicate their response on a five-point scale from “Strongly agree” (= 1) to “Strongly disagree” (= 5), with the midpoint labeled “Neither agree nor disagree” (= 3). Cronbach’s alpha was 0.47, considerably smaller than α = 0.73 reported by Epstein et al. (1996).

*Cognitive Reflection Test.* The original CRT comprises three short math problems, which participants are asked to solve. For example: “A bat and a ball cost $1.10 in total. The bat costs $1.00 more than the ball. How much does the ball cost?” Toplak et al. (2014) extended the CRT by adding four additional problems. We are here using this more recent seven-item version (CRT-7).

*Self-report questions.* Participants were asked the following questions.^1^ All were forced choice “Yes” / “No.”

- Did you think about any of the scenarios we asked you to read during the last two weeks outside of your participation in this research?
- [If “Yes”] Did you change your mind about any of the scenarios as a result?
- Did you discuss any of the scenarios we asked you to read during the last two weeks with other people?
- [If “Yes”] Did you change your mind about any of the scenarios as a result?

#### Participants

All 288 participants who ended up in the analysis of Study 1a were invited to return for a third wave (W3). 262 participants completed W3 within a set time window (6–8 days after completing W2). We excluded 21 participants who failed an instructional attention check (Oppenheimer et al. 2009), so the final sample included *n* = 241 participants (165 female; *M*(*SD*) = 33.1(12.0) y; 27.0% students; 95.9% UK nationals).

#### Procedure

Participants were contacted by Prolific with an invitation to participate in the study, which was hosted on LimeSurvey (https://www.limesurvey.org). As in Study 1a, participants gave informed consent, received instructions, and then completed the scenarios. Next, participants completed the AOT-10, NFC-5, and CRT-7. For each measure, we randomized the presentation order of items. Following this, participants answered a series of self-report questions and provided (additional) demographic information.

Participants were compensated $1.00. An additional bonus of $1.25 was offered and paid to participants who completed all three waves in Studies 1a and 1b.

### Analysis

We again start by calculating correlations, as well as rating shifts, reversals, adoptions and rejections for responses between responses in W2 and W3. Table 3 shows the results.

**Table 3:** Pearson’s product-moment correlation coefficients, proportions and mean magnitudes of rating shifts, reversals, adoptions and rejections for our sacrificial dilemmas between W2 and W3; square brackets show 95% confidence intervals, round brackets standard deviations

	*r*		Shifts	Reversals	Adoptions	Rejections
**Shark**	0.68 [0.61, 0.74]	Prop.	0.44 [0.38, 0.51]	0.17 [0.13, 0.22]	0.05 [0.03, 0.08]	0.05 [0.03, 0.09]
		Mean magnitude	1.9 (1.15)	3 (1.02)	1.55 (0.82)	1.46 (0.66)
**Gas**	0.65 [0.57, 0.72]	Prop.	0.52 [0.46, 0.58]	0.16 [0.12, 0.21]	0.06 [0.04, 0.1]	0.05 [0.03, 0.08]
		Mean magnitude	1.9 (1.29)	3.33 (1.32)	1.6 (0.83)	1.45 (0.69)
**Footbridge**	0.85 [0.81, 0.88]	Prop.	0.39 [0.33, 0.45]	0.07 [0.05, 0.11]	0.04 [0.02, 0.07]	0.04 [0.02, 0.07]
		Mean magnitude	1.5 (0.86)	2.78 (0.94)	1.8 (0.92)	1.33 (0.5)
**Transplant**	0.69 [0.62, 0.75]	Prop.	0.26 [0.21, 0.32]	0.06 [0.03, 0.1]	0.01 [0, 0.04]	0.02 [0.01, 0.04]
		Mean magnitude	1.95 (1.55)	4.21 (1.72)	1.67 (1.15)	2.25 (0.96)
**Expedition**	0.74 [0.68, 0.79]	Prop.	0.5 [0.44, 0.56]	0.15 [0.11, 0.2]	0.05 [0.03, 0.09]	0.04 [0.02, 0.07]
		Mean magnitude	1.74 (1.17)	3.03 (1.34)	1.31 (0.48)	1.33 (0.5)
**Baby**	0.77 [0.72, 0.82]	Prop.	0.46 [0.4, 0.52]	0.1 [0.07, 0.14]	0.04 [0.02, 0.07]	0.1 [0.06, 0.14]
		Mean magnitude	1.71 (1.22)	3.25 (1.62)	1.7 (0.82)	1.61 (0.84)
**Overall**	0.74 [0.68, 0.79]	Prop.	0.43	0.12	0.04	0.05
		Mean magnitude	1.78 (1.21)	3.27 (1.33)	1.6 (0.84)	1.57 (0.69)

In order to investigate whether changes between W1 and W2 tended to persist when a dilemma was judged for a third time (as would be expected if most changes are changes in light of reasons), we fit a pair of binomial mixed-effects models with logit link (Bates et al. 2015). Both models included random intercepts for scenario and participant (Baayen et al. 2008). The first model predicted the occurrence of W2-W3 rating shifts (0 = no rating shift; 1 = rating shift) by the occurrence of W1-W2 rating shifts. There was a moderate effect (*OR* = 3.11 [2.42, 4.01]), such that the odds that a participant would exhibit a rating shift between W2 and W3 were *higher* if they had already shifted between W1 and W2.

The second model predicted the occurrence of W2-W3 rating revisions (0 = no rating revision; 1 = rating revision) by the occurrence of W1-W2 rating revision. There was a moderate effect (*OR* = 4.94 [3.59, 6.85]), such that the odds of a participant exhibiting a rating revision between W2 and W3 were *higher* if they had previously exhibited a revision between W1 and W2.

Our results suggest that changes of moral judgments between W1 and W2 did not generally persist. Instead, participants who changed their judgment between W1 and W2 were *more* likely to change again between W2 and W3. To the extent that moral judgment changes would be expected to persist when they are based on reasons (as we have suggested), these findings speak against the reasons hypothesis.

Next, we looked for a relationship between Actively Open-minded Thinking, Need for Cognition and performance on the Cognitive Reflection Test with changes of moral judgment. We fit a pair of binomial mixed-effects models with logit link, one predicting the occurrence of rating shifts across all three waves by scores on the AOT-10, NFC-5, and CRT-7, the other predicting the occurrence of rating revision across all three waves by scores on the AOT-10, NFC-5, and CRT-7. Because the outcome of these models is the occurrence of both W1-W2 and W2-W3 ratings shifts (reversals), these models only included data from participants who completed all three waves. (The results do not change notably if we restrict the model to W1-W2 rating shifts and also include participants who only completed the first two waves.) To aid interpretation, scores on the AOT-10, NFC-5, and CRT-7 were standardized. Pair-wise correlations between the three scores were small (|*r*| ≤ 0.12), so collinearity does not threaten the interpretation of the results. Both models included random intercepts for participant and scenario.

Scores on the AOT-10, NFC-5 and CRT-7 did not predict the occurrence of rating shifts, in the sense that all 95% CIs include 1.00 (AOT-10: *OR* = 0.89 [0.79, 1.01]; NFC-5: *OR* = 1.11 [0.98, 1.26]; CRT-7: *OR* = 0.92 [0.81, 1.05]). Scores on the AOT-10 also did not predict the occurrence of rating revision (*OR* = 0.96 [0.82, 1.11]). While NFC-5 scores and CRT-7 scores did predict the occurrence of rating revision, both effects are very weak, and in opposite directions (NFC-5: *OR* = 1.18 [1.01, 1.37]; CRT-7: *OR* = 0.84 [0.72, 0.98]). These results fail to support the reasons hypothesis to the extent that Actively Open-minded Thinking, Need for Cognition, and performance on the Cognitive Reflection Test are associated with a tendency to change one’s moral judgments in light of reasons (as we suggested).

Finally, we looked at participants’ responses to our self-report questions. 46.5% [40.3%, 52.8%] of participants reported thinking about (thought) or discussing with others (discussion) one or more of our scenarios outside of the study. 16.2% [12.1%, 21.4%] of participants reported having changed their mind about at least one scenario due to thought or discussion. Surprisingly, not all participants who self-reported changes of mind in light of reasons did in fact change their ratings. Participants who self-reported a change of judgment in light of reasons *and* actually exhibited at least one rating shift accounted for 16.7% [12.4%, 22.0%] of W1-W2 rating shifts, and 16.3% [11.9%, 21.8%] of W2-W3 rating shifts. Participants who self-reported a change of judgment in light of reasons *and* exhibited at least one rating revision accounted for 18.8% [13.8%, 25.1%] of W1-W2 revisions, and 19.0% [13.5%, 25.9%] of W2-W3 revisions. Thus, self-reported changes of mind in light of reasons accounted for only a small proportion of the observed changes (shifts and revisions) in moral judgments. Thus, the reasons hypothesis is again unsupported.

## Discussion

As we have said, philosophers need not be worried by moral instability if it is due to people changing their mind in light of reasons, perhaps found by intervening reflection or discussion with other people. However, Study 1b did not find evidence that, when participants changed their moral judgments in Study 1a, they mostly did so in light of reasons. First, moral judgment changes between W1 and W2 did not generally persist when participants judged the dilemmas for a third time. If fact, participants who exhibited W1-W2 rating changes (shifts, reversals, rejections, and adoptions) were *more* likely to change again between W2 and W3. Second, neither Actively Open-minded Thinking (Baron 1993), nor Need for Cognition (Cacioppo and Petty 1982), nor scores on the Cognitive Reflection Test (Frederick 2005) predicted whether participants changed their moral judgments between waves. Third, participants who self-reported having changed their mind about at least one scenario because they thought more about the scenario or because they discussed the scenario with others accounted for only a small proportion of the moral judgment changes that we in fact observed. These results speak against the reasons hypothesis.

## General Discussion

We have argued that the stability of moral judgments over time is an important feature of moral cognition for philosophers and psychologists to consider. Next, we presented an original empirical study into the stability over 6–8 days of moral judgments about acts in sacrificial dilemmas. Like Helzer et al. (2017, Study 1), we found an overall test-retest correlation of 0.66. Moreover, we observed moderate to large proportions of rating shifts, and small to moderate proportions of rating revisions (*M* = 14%), rejections (*M* = 5%) and adoptions (*M* = 6%)—that is, the participants in question judged *p* in one wave, but did not judge *p* in the other wave.

### What Explains Instability?

One potential explanation of our results is that they are not a genuine feature of moral judgments about sacrificial dilemmas, but instead are due to measurement error. Measurement error is the difference between the observed and the true value of a variable. So, it may be that most of the rating changes we observed do not mean that many real-life moral judgments about acts in sacrificial dilemmas are (or would be) unstable over short periods of time. Instead, it may be that when people make moral judgments about sacrificial dilemmas in real life, their judgments remain very stable from one week to the next, but our study (perhaps any study) was not able to capture this stability.

To the extent that real-life moral judgment is what moral psychologists and philosophers are interested in, this may suggest a problem with the type of study design used in this and many other papers. If there is enough measurement error, then it may be very difficult to draw firm conclusions about real-life moral judgments from this research. Other researchers have raised related objections. Most forcefully, Bauman et al. (2014) have argued that participants often do not take the judgment tasks used by moral psychologists seriously enough for them to engage with these tasks in anything like the way they would if they came across the same tasks in the real world (also, see, Ryazanov et al. 2018). In our view, moral psychologists would do well to more frequently move their studies outside of the (online) lab and into the real world (e.g., Bollich et al. 2016; Hofmann et al. 2014).

How much measurement error would be too much? We have already mentioned that psychometricians often consider test-retest correlations of 0.70 and above adequate. By this measure, because the 95% confidence interval of our overall correlation coefficient includes this value, our results may be considered good news for moral psychologists (cf., Helzer et al. 2017, p. 4). Then again, looking at our scenarios individually, for half of them, the evidence is consistent with test-retest correlations below 0.70. Moreover, different contexts allow for different levels of measurement error. If you want to build a garden shed, measuring your building materials to within a centimeter or so before cutting is likely good enough; the same is not true if you want to build a spaceship. We think that there are at least some contexts in which our results may be more worrying. For example, researchers sometimes use single sacrificial dilemmas to measure trait utilitarianism and deontology (e.g., Bègue and Laine 2017; Côté et al. 2013). Our Study 1a suggests that in these studies, about 1 in every 7 participants would test one way one week, but a different way the next. This level of error does not strike us as acceptable.

Instead, our findings may tell us something about a genuine feature of real-life moral judgment. If so, then a natural question to ask is what makes moral judgments unstable (or stable) over time. In this paper, we have looked at three possible explanations, but we did not find evidence for them. First, because sacrificial dilemmas are in a certain sense designed to be difficult, moral judgments about acts in these scenarios may give rise to much more instability than moral judgments about other scenarios or statements. However, when we compared our test-retest correlations with a sampling of test-retest correlations from instruments involving other moral judgments, sacrificial dilemmas did not stand out. Second, we did not find evidence that moral judgment changes occur because people are more confident in their moral judgments the second time around. Third, Study 1b did not find evidence that rating changes, when they occurred, were often due to changes in light of reasons and reflection. Note that this does not mean that we can rule out any of these potential explanations for unstable moral judgments completely. As we point out below, our research is limited in the extent to which it could test each of these explanations, and so one or more of them may still have been the cause for some proportion of the rating changes we observed.

Another possible explanation that we did not investigate is that instability may be due to the influence of situational influences. There are variety of possible candidates for such influences (for a review, see, Klenk 2021), including incidental emotions (e.g., Strohminger et al. 2011; Valdesolo and DeSteno 2006), framing effect (for a review, see, Rehren and Sinnott-Armstrong 2021), and social conformity (for a review, see, Chituc and Sinnott-Armstrong 2020). The thought is that for many, perhaps all of our participants, their environment, circumstances or state of mind were different when they participated in the first wave of our study compared to when they participated in the second wave. Because of this, so goes the explanation, the moral judgments of some of our participants were affected by one set of situational factors in one wave, but by another set of situational factors in the next wave—enough for the participant’s moral judgments to change across the two waves. For examples, participants may have made different moral judgments in different waves because in one of the waves, they were tired (Killgore et al. 2007; Kouchaki and Smith 2014; but, see, Tempesta et al. 2012; Trémolière and Gosling 2021). Or drunk (Duke and Bègue 2015; but, see, Paruzel-Czachura et al. 2021). Or their computer was next to a smelly trash can (Schnall et al. 2008; but, see, Ugazio et al. 2012).

In addition to what makes moral judgments unstable, there is also the question of who makes unstable moral judgments. One option is that there are individual differences between people, such that some people are much more prone than other people to make unstable moral judgments. In terms of a potential influence of distorting factors on moral judgments, people may differ in how much these factors affect their moral cognition. Another option (though it does not exclude the first option) is that people generally make stable moral judgments, but from time to time, instability (perhaps in the form of the distorting influence of situational factors) creeps in for everyone.

There is some evidence in favor of this second explanation: First, we and Helzer et al. (2017, Study 1) both find that the overall pattern of people’s moral judgments about acts in a series of sacrificial dilemmas tends to be quite stable across short periods of time. A plausible explanation for this finding is that most of the distorting influences that may affect moral judgments about single sacrificial dilemmas average out across multiple sacrificial dilemmas. Second, when we investigated whether the rating changes that we observed were due to only some participants changing their rating on many scenarios, or instead to most participants changing their rating on a small number of scenarios, our results suggest that the rating changes we observed were not generally due to a few participants changing their rating on many scenarios, but instead were due to many participants changing their rating on a few scenarios.

## Philosophical Implications

Our findings of instability without reason also may raise serious questions for moral philosophy, including normative moral theory, applied ethics, meta-ethics, and virtue theories.

As we said, many moral philosophers treat moral judgments about specific cases like sacrificial dilemmas as part of the “data of ethics” (Ross 1930/2002, p. 41) when they use these judgments to choose among competing normative moral theories (e.g., see, Kamm 1993; Rawls 1971). However, this data is unreliable when moral judgments change in the ways we observed, because incompatible moral judgments about the same act in the same circumstances cannot both be correct. Such a shifting foundation seems not to be a good place to build a moral theory—or a house (Matthew 7:26)—if you want them to last (see, e.g., Alexander et al. 2010/2014; Horowitz 1998).

Instability also challenges the most common methods in applied ethics. For example, if many COVID-19 patients need a ventilator, but only one is available, then bioethicists might argue for a moral judgment that a certain patient rather than the others should receive the ventilator. This real case is comparable in some ways to the sacrificial dilemmas we studied. But if people are likely to make an incompatible moral judgment a few days later without any new information or reason to change, then it is hard to see why they would be justified in trusting the moral judgment that they happen to make on a given day. If we have no independent way to tell which of the two moral judgments is correct, then we cannot determine which one—if either—is trustworthy.

Instability also creates trouble for the meta-ethical theory known as intuitionism (e.g., Audi 2007; Huemer 2005; Ross 1930/2002). A popular view of moral intuitions is that they are moral judgments that are not based on any other beliefs or judgments. Moral intuitionists argue that some moral intuitions are justified even if the person who has those intuitions is not able to give any argument to support them. One common argument for this claim is that the process that produces moral intuitions can be reliable even when the person with the moral intuition does not understand why it is reliable and cannot argue that it is reliable (e.g., Shafer-Landau 2005). However, if people have incompatible moral intuitions on different days for no good reason, then the process that produces moral intuitions cannot be reliable. If so, then instability without reason deprives moral intuitionists of a popular argument for their view and raises serious doubts about whether moral intuitions can be justified independently of inference (see, Sinnott-Armstrong, 2008a).

Finally, most moral virtue theories assume that people do not have the virtue of honesty, for example, unless they both do the honest act and also do it because they judge it to be honest or right (e.g., Miller 2017). But then, if people make incompatible moral judgments a few days later without any reason, they cannot continue to have the virtue of honesty on both days. This instability is a problem for virtue theories that see moral virtues as character traits that remain constant over time (Aristotle 2019). Virtue theorists might reply that virtues are ideals that few, if any, of us achieve (e.g., Sreenivasan 2017), but it strikes many others as implausible for moral theories to be built on requirements or ideals that almost no humans can fulfill (e.g., Flanagan 1991).

Philosophers might reply that, even if the moral judgments of participants in our study were unstable to some degree, they were not completely unstable. Perhaps most relevant, almost half (46%) of participants never reversed their ratings on a single scenario. Why is this observed stability not enough? What degree of stability is required for moral theories to have solid foundations, for applied ethics to be trustworthy, for moral intuitions to be justified, and for actual people to have moral virtues? Any answer is bound to be controversial. Still, it is widely assumed that a higher degree of reliability is required when mistakes are more costly. My airplane needs to be more reliable than my skateboard needs to be. In addition, mistakes in moral judgments can be very costly, such as in a sacrificial dilemma where lives are at stake. These assumptions imply reason to require a high level of reliability in moral judgments about serious cases, though it is still not clear how high is high enough.

A final response is that, even if the moral judgments of our participants are unreliable, moral philosophers and other experts in ethics still might do better. Although this is possible, there is evidence that speaks against it. Some recent studies have found that several morally irrelevant influences on lay moral judgments also recur in the moral judgments of moral philosophers (e.g., Schwitzgebel and Cushman 2012, 2015; Tobia et al. 2013). Thus, our findings that lay moral judgments exhibit instability without reason provide at least some reason to suspect that professional moral judgments might not be as stable as their proponents assume. If so, then even expert moral judgments might be too unstable for solid moral theories, trustworthy applied ethics, or justified moral intuitions. Experts, including moral philosophers, might be better at moral judgment than the participants in our studies, but that remains to be shown.

Scientific studies of the stability over time of moral judgments can, thus, be illuminating for several central issues is moral philosophy. These lessons for moral philosophy and for moral psychology show some of the ways in which our findings in the reported studies are important, even if they do not finally settle any of these controversial issues.

## Limitations and Future Research

This research is subject to several limitations. First, in this study, we only looked at moral judgments about acts in a small set of sacrificial dilemmas. This limits the generalizability of our results in (at least) two ways. One, many of the scenarios we used may be too unrealistic to allow us much insight into real-life moral judgments about situations involving sacrificial dilemmas (see, e.g., Bauman et al. 2014; Bostyn et al. 2018; Kneer and Hannikainen 2022). Two, focusing on sacrificial dilemmas means that we cannot draw conclusions about the stability of other moral judgments. For example, moral judgments can involve different moral concerns from harm (e.g., fairness, honor, honesty; see, Haidt and Graham 2007), different groups of people instead of strangers (e.g., family, friends, work colleagues; see, Bloom 2011), and different levels of severity than death (e.g., for harm: assault, property damage, insults). Future research should investigate these other kinds of moral judgments.

Second, many of the potential philosophical implications we have discussed assume that our study sheds light on the stability (or instability) of the moral judgments that professional philosophers and other experts make as part of their work. However, we did not sample from these groups for this study, and so we cannot be sure that the assumption is justified. It may that the moral judgments of professional philosophers and other experts are much more (or less) stable than the moral judgments of lay people, perhaps because of their special training in areas like ethical theory and moral reasoning. Future research should investigate these issues in professional philosophers and other experts in ethics.

Third, rating revisions are likely the clearest evidence of instability. If so, then future studies about this topic may consider using a dichotomous scale to measure moral judgments instead of the seven-point scale we used. This would help to remove the interpretative difficulties associated with dichotomizing ratings obtained on a continuous scale, for example, how to interpret ratings that are only a single point off of the scale midpoint. We interpret such ratings as contentful moral judgments (for example, “leaning towards should not do it” or “the act is immoral to some extent”), but it may be that they are more adequately interpreted as participants basically being undecided.

Fourth, while we strove to provide a reasonably solid test of the reasons hypothesis in Study 1b by making use of several different operationalizations, our test is still not air-tight. For example, because people’s introspective access into their own judgment and decision-making is frequently inaccurate (Nisbett and Wilson 1977; Wilson and Dunn 2004), there is doubt about the evidential weight of our self-report questions. Also, our NFC-5 had poor internal consistency, meaning that the results involving it need to be taken with a large grain of salt. One way to do better would be to directly manipulate reflective engagement with the dilemmas. For example, participants could be asked to read a series of arguments against their judgments on certain dilemmas between waves (cf., Stanley et al. 2017). Future research along these lines could provide more decisive evidence for or against the reasons hypothesis.

Fifth, in addition to the reasons hypothesis, we also probed two other potential explanations. First, to find out whether sacrificial dilemmas give rise to more unstable moral judgments than other scenarios or statements, we compared our results with a sampling of test-retest correlations from instruments involving other moral judgments. However, the other studies included in this comparison differ from ours in multiple respects (e.g., different rating scales; longer delay between waves), which limits the confidence one should have in this comparison. Future research should explore the stability of moral judgments about different types of stimuli (sacrificial dilemmas, moral statements, etc.) in the same sample of participants.

Sixth, to find out whether most rating changes occur because people are more confident in their moral judgments the second time around, we analyzed whether participants tended to give more extreme ratings in the second wave than in the first wave. However, it is not clear that more extreme rating can always be interpreted as more confident ratings, and so our study can only shed limited light on this explanation. A straight-forward way to do better would be to ask participants for a confidence rating, in addition to their moral judgment.

Finally, our study did not test other potential explanations for instability, such as the influence of situational factors on moral judgment. One way for future research to investigate this would be to record (for example, via self-report) the presence or absence of different likely situational influences in the situation when people make moral judgments, and then to find out whether this predicts moral instability. We encourage such research.

## Data Availability

The study materials and data for all studies reported in this paper are available at: https://osf.io/tfqe6/.

## Appendix

### Sacrificial dilemmas

Participants rated all scenarios on a seven-point rating scale, from “Definitely should do it” (= 1) to “Definitely should not do it” (= 7); the scale-midpoint (= 4) was labeled “Neither.”

#### Shark

[Agent] is visiting the beach. He is walking along a pier when he notices a shark swimming towards the beach. [Agent] can see that this shark will attack and kill everyone in the first group of swimmers it encounters. Directly in the path of the shark is a group of five swimmers. The shark is too fast for them to get out of the water in time. There is one swimmer in the water between [agent] and the shark.

[Agent] realizes that he can make loud noises that he knows will attract the shark, diverting it from its current path. If [agent] does not make loud noises, the shark will attack the five swimmers in its path, but not the one swimmer in the water between him and the shark. If [agent] makes loud noises, the shark will kill the one swimmer in the water between him and the shark, but not the five swimmers in its original path.

Should [agent] make loud noises?

#### Gas

[Agent] is visiting a chemical plant when she notices that there is gas leaking into a room close by. [Agent] can see that this gas is poisonous and will kill anyone it comes into contact with. There are five workers in the room. They are unaware of the gas and will not be able to get out of the room in time. Nearby, there is a second, smaller room with one worker in it.

[Agent] realizes that there is a switch next to her, which she can push to direct the gas away from the room with the five workers and into the smaller room with one worker. If [agent] does not push the switch, the gas will kill the five workers, but not the one worker. If [agent] pushes the switch, the gas will kill the one worker, but not the five workers.

Should [agent] push the switch?

#### Footbridge

[Agent] is standing on a footbridge across the railroad tracks when he notices an empty railroad car out of control. [Agent] can see that this railroad car is moving so fast that it will kill anyone it hits. On the track ahead are five people. The banks are so steep that they will not be able to get off the track in time. On the bridge with [agent] is a large man wearing a backpack.

[Agent] realizes that he can push the large man off the footbridge onto the railroad tracks in front of the railroad car that is out of control. He knows that the weight of the large man and his backpack would be sufficient to stop the railroad car. If [agent] does not push the large man off the footbridge, the railroad car will kill the five people on the track, but not the large man on the footbridge. If [agent] pushes the large man off the footbridge, the railroad car will kill the large man, but not the five people on the track.

Should [agent] push the large man off the footbridge?

#### Transplant

[Agent] is a surgeon in a hospital. She has five patients suffering from different organ failures. All five patients are in critical condition and will die if they do not get an immediate organ transplant. [Agent] is currently performing a routine surgery on a sixth patient who is otherwise healthy. From reading the patient’s file, [agent] knows that this patient’s organs are compatible with the five other patients.

[Agent] realizes that she can cut the sixth patient’s main artery during surgery. This will kill that patient, but will make the required transplants available for the five patients suffering from organ failure. If [agent] does not cut the one patient’s main artery, this patient will live, but the five patients will die. If [agent] cuts the one patient’s main artery, this will kill that patient, but the five patients will live.

Should [agent] cut the patient’s main artery?

#### Expedition

[Agent] is part of a group of six ecologists who are studying wildlife in a remote stretch of jungle. The group has been taken hostage by a group of paramilitary terrorists. [Agent] understands the terrorist’s language and knows that their leader will kill him and the other five hostages the following morning. The hostages are guarded day and night and will not be able to escape.

One of the guards has taken a liking to [agent]. He informs him that he is willing to help [agent] and the other hostages escape. However, as an act of good faith, he wants [agent] to cut the throat of one of his fellow hostages whom the guard does not like. If [agent] does not cut the throat of the fellow hostage, [agent] and the remaining five hostages will be killed the next morning. If [agent] cuts the throat of the fellow hostage, this will kill that hostage, but [agent] and the remaining four hostages will not be killed the next morning.

Should [agent] cut the throat of the fellow hostage?

#### Baby

[Agent] lives in a small village that has been taken over by enemy soldiers. [Agent] understands the enemy soldiers’ language and knows that they are under orders to kill every villager they find. [Agent] is hiding out in the cellar of a large house with her baby and four neighbors. Upstairs, they can hear the voices of soldiers who have come to search the house for valuables. [Agent]’s baby begins to cry loudly. [Agent] knows that this will attract the attention of the soldiers. The cellar only has one exit, so the group will not be able to escape.

[Agent] realizes that she can press her hand over her baby’s mouth to stop the crying. However, she knows that this will cut off the baby’s breath, ultimately suffocating and killing it. If [agent] does not press her hand over the baby’s mouth, [agent], the baby, and the four neighbors will all be killed by the enemy soldiers. If [agent] presses her hand over the baby’s mouth, this will kill the baby, but [agent] and her four neighbors will not be killed by the enemy soldiers.

Should [agent] press her hand over the baby’s mouth?

## Footnotes

1 Participants were asked several other self-report questions. Since they do not bear on the issues discussed in this paper, we do not include them in the main text. The questions and participants’ responses are available at: https://osf.io/tfqe6/.

## Code Availability

The R analysis code necessary to reproduce all analyses reported in the paper is available at: https://osf.io/tfqe6/.
